# Peripheral immune-derived matrix metalloproteinase promotes stress susceptibility

**DOI:** 10.21203/rs.3.rs-1647827/v1

**Published:** 2023-01-30

**Authors:** Flurin Cathomas, Hsiao-Yun Lin, Kenny L. Chan, Long Li, Romain Durand-de Cuttoli, Lyonna F. Parise, Antonio V. Aubry, Samer Muhareb, Fiona Desland, Yusuke Shimo, Aarthi Ramakrishnan, Molly Estill, Carmen Ferrer-Pérez, Eric M. Parise, Jun Wang, Allison Sowa, William G. Janssen, Sara Costi, Adeeb Rahman, Nicolas Fernandez, Filip K. Swirski, Eric J. Nestler, Li Shen, Miriam Merad, James W. Murrough, Scott J. Russo

**Affiliations:** 1 Nash Family Department of Neuroscience, Brain & Body Research Center, Icahn School of Medicine at Mount Sinai, New York, NY, USA.; 2 Friedman Brain Institute, Icahn School of Medicine at Mount Sinai, New York, NY, USA.; 3 Department of Oncological Sciences, Precision Immunology Institute, Tisch Cancer Institute, Icahn School of Medicine at Mount Sinai, NY, USA.; 4 Microscopy CoRE and Advanced Bioimaging Center, Icahn School of Medicine at Mount Sinai, New York, NY, USA.; 5 Depression and Anxiety Center for Discovery and Treatment, Department of Psychiatry, Icahn School of Medicine of Mount Sinai, New York, NY, USA.; 6 Cardiovascular Research Institute, Icahn School of Medicine at Mount Sinai, New York, NY, USA.

## Abstract

Psychosocial stress has profound effects on the body, including the peripheral immune system and the brain^1,2^. Although a large number of pre-clinical and clinical studies have linked peripheral immune system alterations to stress-related disorders such as major depressive disorder (MDD)^3,4,5^, the underlying mechanisms are not well understood. Here we show that a peripheral myeloid cell-specific proteinase, matrix metalloproteinase 8 (MMP8), is elevated in serum of subjects with MDD as well as in stress-susceptible (SUS) mice following chronic social defeat stress (CSDS). In mice, we show that this increase leads to alterations in extracellular space and neurophysiological changes in the nucleus accumbens (NAc), thereby altering social behaviour. Using a combination of mass cytometry and single-cell RNA-sequencing, we performed high-dimensional phenotyping of immune cells in circulation and brain and demonstrate that peripheral monocytes are strongly affected by stress. Both peripheral and brain-infiltrating monocytes of SUS mice showed increased **Mmp8** expression following CSDS. We further demonstrate that peripheral MMP8 directly infiltrates the NAc parenchyma to control the ultrastructure of the extracellular space. Depleting MMP8 prevented stress-induced social avoidance behaviour and alterations in NAc neurophysiology and extracellular space. Collectively, these data establish a novel mechanism by which peripheral immune factors can affect central nervous system function and behaviour in the context of stress. Targeting specific peripheral immune cell-derived matrix metalloproteinases could constitute novel therapeutic targets for stress-related neuropsychiatric disorders.

Stress-related neuropsychiatric disorders such as major depressive disorder (MDD) have a high worldwide prevalence and tremendous individual burden^6^. While there are many effective treatments for MDD, more than a third of affected individuals do not achieve full remission by available antidepressant medications or established psychotherapeutic treatments^7,8^. One of the most important risk factors for depression is chronic psychosocial stress^9^. Therefore, elucidating the pathophysiological mechanisms underlying the effects of psychosocial stress is crucial to advancing our understanding of disorders like MDD and ultimately develop treatment options and prevention strategies.

Immune interactions between the central nervous system and peripheral organ systems are tightly regulated^10^. Psychosocial stress can profoundly impact this bi-directional communication, and disrupted neuroimmune interactions are increasingly recognized as important factors in the pathogenesis of stress disorders^11^. Chronic stress activates the innate immune system resulting in mobilization of peripheral myeloid cells (e.g., monocytes and neutrophils) and the production of pro-inflammatory cytokines, such as interleukin-6 (IL-6)^12,13,14^ In humans, it is well established that a subset of patients with stress-related neuropsychiatric disorders, such as MDD, display a state of chronic low-grade inflammation, characterized by increased circulating pro-inflammatory cytokines and leukocytosis^4,14,15^. In addition to these peripheral immune changes, stress disrupts the endothelial blood-brain barrier (BBB) in mice allowing greater entry of circulating proteins directly into brain reward regions like the nucleus accumbens (NAc)^16,17^ While these findings have provided important insights into the pathophysiology of stress and depression, we still know relatively little about the mechanisms by which these stress-induced immune changes affect neuronal function and ultimately behaviour.

In the brain, neurons and non-neuronal cells are separated by the extracellular space which contains interstitial fluid and the extracellular matrix (ECM), a dense scaffold of proteins and glycans secreted by neurons and glial cells^18,19^. ECM molecules have been shown to play an important role in homeostatic processes of the brain, including synaptic function^20^. ECM degradation and remodeling is regulated by various enzymes, such as Matrix metalloproteinases (MMPs)^19,21^. MMPs in circulation have been associated with numerous inflammatory processes and disorders, such as cancer and myocardial infarction^22,23^. While several studies have implicated central nervous system MMPs in synaptic remodeling and transmission by altering components of the ECM^24,25,26^, little is known about the effects of peripheral immune-derived MMPs in the context of psychosocial stress.

## Chronic stress and pro-inflammatory monocytes

**Extended Data Fig. 1.**
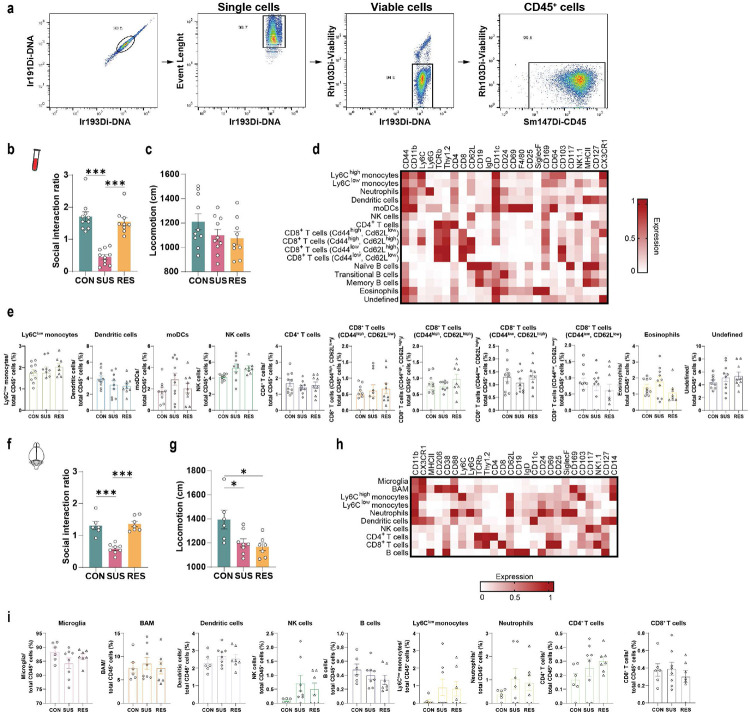
High-dimensional phenotyping of immune cells in brain and blood with CyTOF (Cytometry by time-of-flight). **a,** Gating strategy for live CD45^+^ cells. **b,** Social interaction ratio and **c,** locomotion of unstressed control (CON), susceptible (SUS) and resilient (RES) mice (behavioural data from blood CyTOF experiment). **d,** Marker expression and assigned leukocyte subpopulations in blood. **e,** Leukocyte subpopulation frequencies in blood. **f,** Social interaction ratio and **g,** locomotion of CON, SUS and RES mice (behavioural data from brain CyTOF experiment). **h,** Marker expression and assigned leukocyte subpopulations of CD45^+^ brain leukocytes. **i,** Leukocyte subpopulation frequencies in brain. (One-way ANOVA with Bonferroni *post hoc* test; for blood, each data point represents one biological sample; for brain, each data point represents four pooled brains). * p < 0.05, *** p < 0.001. Data are shown as means ± s.e.m. Abbreviations: moDCs: Monocyte-derived dendritic cells; NK cells: Natural killer cells; BAM: Border-associated macrophages.

**Fig. 1.**
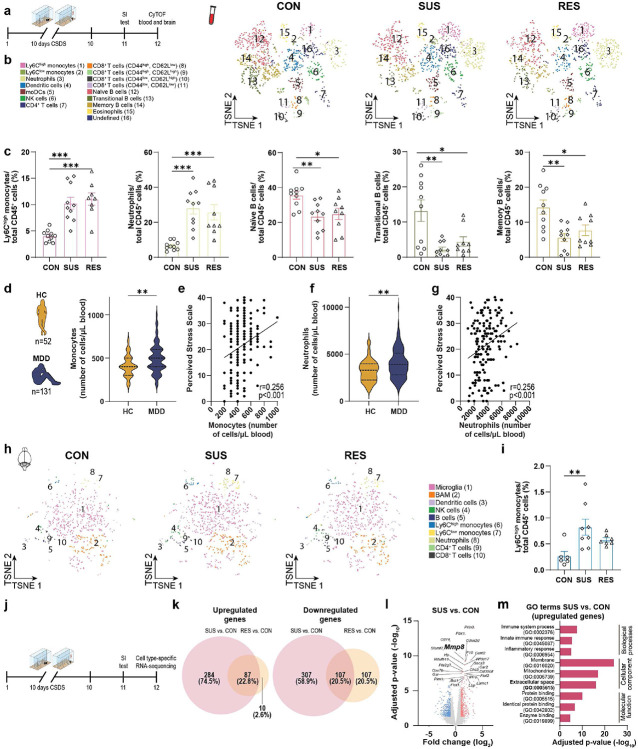
Stress increases monocyte numbers in circulation and in the brain, and induces a proinflammatory transcriptional signature in stress-susceptible (SUS) mice. **a,** Experimental outline: After 10 days of chronic social defeat stress (CSDS), mice were tested in the social interaction (SI) test. Leukocyte subpopulation frequencies were determined in blood and brain of unstressed control (CON), SUS and resilient (RES) mice using mass cytometry (Cytometry by time-of-flight, CyTOF). t-distributed stochastic neighbor embedding (t-SNE) maps of CD45^+^ cells in **b,** blood and **h,** brain. The color of each cluster corresponds to the assigned cell type. **c,** Frequencies of Ly6C^high^ monocytes, neutrophils and B cells in circulation in CON, SUS and RES mice. (One-way ANOVA with Bonferroni *post hoc* test, each data point represents one biological sample). Number of **d,** monocytes and **f,** neutrophils in circulation in patients with major depressive disorder (MDD) compared to healthy controls (HC) (two-tailed Student’s t-test). Correlation between perceived stress (assessed by the Perceived Stress Scale) and **e,** monocyte numbers and **g,** neutrophil numbers (Pearson correlation). **i,** Ly6C^high^ monocytes in whole brain. (One-way ANOVA with Bonferroni *post hoc* test; each data point represents four pooled brains). **j,** Experimental outline of cell type-specific RNA-sequencing of Ly6C^high^, Ly6C^low^ monocytes, B cells and T cells from blood. **k,** Venn diagrams displaying the number of differentially expressed genes (adjusted p-value < 0.05 and log_2_ fold change > ∣1∣) in Ly6C^high^ monocytes. **l,** Volcano plot showing the 25 most significantly differently expressed protein coding genes in Ly6C^high^ monocytes from SUS vs. CON mice. **m,** Top three gene ontology (GO) terms from significantly upregulated genes in Ly6C^high^ monocytes of SUS vs. CON mice. * p < 0.05, ** p < 0.01, *** p < 0.001. Data are shown as means ± s.e.m (panels c and i). Abbreviations: moDCs: Monocyte-derived dendritic cells; NK cells: Natural killer cells; BAM: Border-associated macrophages.

To investigate the effects of psychosocial stress on the immune system and its impact on the brain, we used the chronic social defeat stress (CSDS) paradigm^27,28^. Interpersonal conflicts and social bullying are commonly experienced major psychological stressors that can precipitate a major depressive episode^29,30^. The CSDS paradigm - one of the best validated mouse models of psychosocial stress – consists of the experimental mice being subordinated by an aggressive CD-1 mouse through a combination of physical contact and sensory exposure over 10 days. Despite undergoing the same stress, the stress-response of individual mice varies: a majority of stressed mice develop a behavioural phenotype characterized by social avoidance and anhedonia (stress-susceptible [SUS mice]), while a subset of animals shows behaviours similar to unstressed control (CON) mice and are termed resilient (RES). We first performed high-dimensional phenotyping of immune cells of CON, SUS and RES mice from circulation and brain using mass cytometry (cytometry by time-of-flight, [CyTOF]) (Fig. 1a, Extended Data Fig. 1a). Based on previous studies^31,32^, we compiled a panel of surface-receptor antibodies to capture the major immune cell lineages (**Supplementary Table 1**). In the blood, CSDS led to an increase in inflammatory Ly6C^high^ monocytes and neutrophils, and a decrease in B cells in both SUS and RES mice (Figs. 1b, c, Extended Data Figs. 1b-e; detailed statistical information for each experiment can be found in **Supplementary Table 2**).

**Extended Data Fig. 2.**
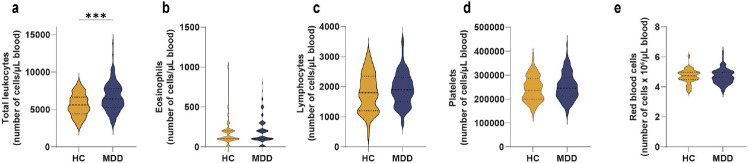
Leukocyte subpopulation frequencies in patients with major depressive disorder (MDD) compared to healthy controls (HC). Number of **a,** total leukocytes, **b,** eosinophils, **c,** lymphocytes, **d,** platelets and **e,** red blood cells in patients with MDD compared to HC. (Two-tailed Student’s t-test [all panels]). *** p < 0.001.

To investigate if these pre-clinical mouse findings also translate to human stress disorders, we assessed leukocyte subpopulations in blood from patients with MDD and healthy controls (HC) and found that patients with MDD displayed leukocytosis driven by increased numbers of monocytes and neutrophils (Figs. 1d, f, Extended Data Figs. 2a-e, **Supplementary Table 3**). We also observed a significant positive correlation between the number of monocytes and neutrophils in circulation with perceived stress, using the Perceived Stress Scale^33^, a clinically validated self-report measure of stress (Figs. 1e, g).

In whole brains of mice following CSDS, we observed a specific increase of infiltrating pro-inflammatory Ly6C^high^ monocytes in SUS, but not RES, mice compared to CON mice (Figs. 1h, i, Extended Data Figs. 1f-h). To prevent contamination with circulating leukocytes, brains were thoroughly perfused with PBS. Notably, we did not observe differences in other infiltrating leukocytes or brain-resident immune cells, such as microglia or border-associated macrophages (Extended Data Fig. 1i).

**Extended Data Fig. 3.**
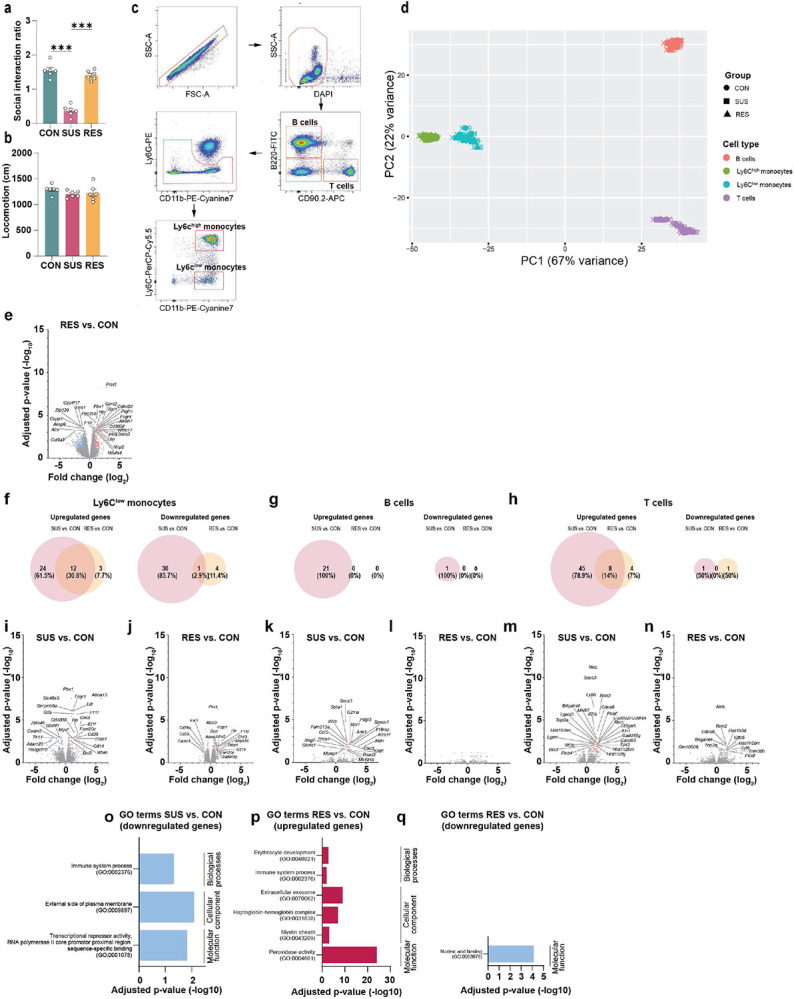
Cell type-specific RNA-sequencing of leukocyte subpopulations in circulation. **a,** Social interaction ratio and **b,** locomotion of unstressed control (CON), susceptible (SUS) and resilient (RES) mice (One-way ANOVA with Bonferroni *post hoc* test). **c,** Representative flow cytometry plots showing gating strategy for fluorescence-activated cell sorting of Ly6C^high^ monocytes, Ly6C^low^ monocytes, B cells and T cells. **d,** Principal component analysis (PCA) plot performed on the top 2000 most variable genes of sorted cells. **e,** Volcano plot showing the 25 most significantly differently expressed protein coding genes in Ly6C^high^ monocytes of RES vs. CON mice. Venn diagrams displaying number of differentially expressed genes (adjusted p-value < 0.05 and log_2_ fold change > ∣1∣) in **f,** Ly6C^low^ monocytes, **g,** B cells and **h,** T cells. Volcano plots showing the 25 most significantly differentially expressed protein coding genes (if applicable) in Ly6C^low^ monocytes [**i,** SUS vs. CON, **j,** RES vs. CON], B cells [**k,** SUS vs. CON, **l,** RES vs. CON] and T cells [**m,** SUS vs. CON, **n,** RES vs. CON]. Top three gene ontology (GO) pathways (if applicable) in Ly6C^high^ monocytes from **o,** significantly downregulated genes of SUS vs. CON mice, **p,** significantly upregulated genes in RES vs. CON and **q,** significantly downregulated genes in RES vs. CON mice. *** p < 0.001. Data are shown as means ± s.e.m.

To investigate differences in stress-induced transcriptional changes in the major circulating leukocyte subpopulations from CON, SUS and RES mice, we performed cell type-specific RNA-sequencing of Ly6C^high^ and Ly6C^low^ monocytes, B cells and T cells (Fig. 1j, Extended Data Figs. 3a-d). CSDS-induced changes in gene expression were most pronounced in Ly6C^high^ monocytes, with a total of 785 differentially expressed genes in SUS vs. CON mice and 311 genes in RES vs. CON mice (adjusted p-value < 0.05 and log_2_ fold change > ∣1∣) (Figs. 1k, l, Extended Data Fig. 3e), with approximately 10 times fewer differentially expressed genes in the other cell types (Extended Data Figs. 3f-q). We then performed gene ontology (GO) enrichment analysis of biological processes, cellular components and molecular function. Genes upregulated in SUS vs. CON mice were involved in GO biological processes such as innate immune response (GO:0045087) and inflammatory response (GO:0006954) and cellular components like extracellular space (GO:0005615) (Fig. 1m). Taken together, CSDS increased monocyte numbers in circulation and in the brain and induced a pro-inflammatory transcriptional signature in SUS mice. These findings, which emphasize the role of peripheral myeloid cells in stress-linked disorders such as MDD, are in line with several pre-clinical studies^13,34,12,35^ and studies in humans^36,37,38^. Therefore, we focused on elucidating the mechanisms by which peripheral monocytes can affect neuronal function and behaviour.

## Brain-infiltrating monocytes express increased matrix metalloproteinase 8 (*Mmp8*)

**Extended Data Fig. 4.**
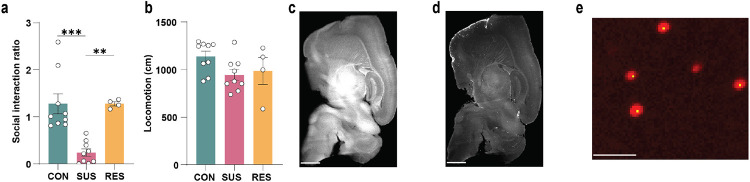
Anatomical mapping of brain-infiltrating *Ccr2*^rfp+^ monocytes using iDISCO+. **a,** Social interaction ratio and **b,** locomotion of unstressed control (CON), susceptible (SUS) and resilient (RES) mice (One-way ANOVA with Bonferroni *post hoc* test). Representative images from lightsheet imaging of **c,** auto-fluorescent signal (scale bar: 1000 μm) and **d,** monocyte signal (scale bar: 1000 μm). **e,** brain-infiltrating monocytes (red) in the nucleus accumbens showing accurate cell detection (yellow) by the automated software Clearmap (scale bar: 30 μm). ** p < 0.01, *** p < 0.001. Data are shown as means ± s.e.m.

**Fig. 2.**
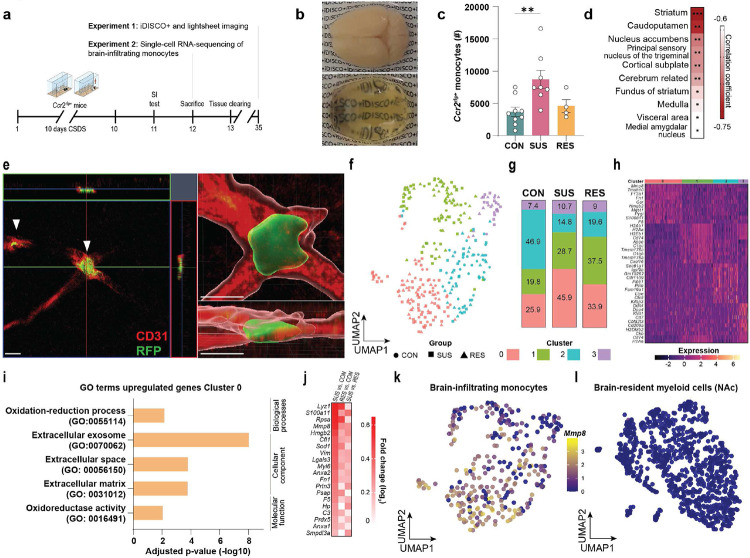
Increased expression of matrix metalloproteinase 8 (*Mmp8*) in brain-infiltrating Ly6C^high^ monocytes of susceptible (SUS) mice. **a,** Outline of iDISCO+ brain clearing and light sheet imaging, and single-cell RNA-sequencing experiments. **b,** Representative images of a mouse brain before (upper image) and after (lower image) iDISCO+ clearing. **c,** Number of *Ccr2*^rfp+^ monocytes in whole brain of control (CON), SUS and resilient (RES) mice (One-way ANOVA with Bonferroni *post hoc* test). **d,** Top ten most significant correlations between brain-infiltrating monocytes and social interaction ratio (lower correlation coefficients (red) indicate that higher numbers of infiltrating monocytes are associated with greater social avoidance) (Pearson correlation). **e,** Reconstruction of immunofluorescence images in the nucleus accumbens (NAc), blood vessels (red), *Ccr2*^rfp+^ monocytes (green, white arrows). Scale bar: 10 μm. **f,** Uniform Manifold Approximation and Projection (UMAP) representation of 4 clusters identified using Seurat clustering. **g,** Relative (%) abundance of number of cells per cluster in CON, SUS and RES mice. **h,** Heat map of top ten cluster-defining protein coding genes. **i,** Gene ontology (GO) terms of significantly (adjusted p-value < 0.05) upregulated genes of Cluster 0. **j,** Genes of GO terms “extracellular space” and “extracellular matrix” compared between SUS vs. CON, RES vs. CON and SUS vs. RES mice. Feature plots of normalized gene expression of *Mmp8* in **k,** brain-infiltrating *Ccr2*^rfp+^ monocytes and **l,** brain-resident immune cells in the NAc. ** p < 0.01. Data are shown as means ± s.e.m.

First, we sought to investigate the exact locations where infiltrating inflammatory Ly6C^high^ monocytes traffic in the brain. We performed detailed anatomic mapping of brain-infiltrating monocytes with the whole brain tissue clearing method iDISCO+^39^ Using the *Ccr2*^rfp^ reporter line, where monocytes express a red fluorescent protein, we cleared brains from CON, SUS and RES mice, then performed lightsheet microscopy and registered the samples to the Allen Brain Atlas using ClearMap^39^ (Figs. 2a, b, Extended Data Figs. 4a-e). We first analysed total infiltrating cells in whole brains and confirmed our CyTOF data showing increased monocytes only in SUS mice (Figs. 1i and 2c). Next, we examined the correlation between infiltrating monocytes and social avoidance behaviour. Cell counts in striatal regions such as the NAc, were highly correlated with the social interaction (SI) ratio of stressed mice, with higher numbers of NAc-infiltrating monocytes correlating with greater social avoidance behaviour (Fig. 2d). The NAc is a stress-responsive brain region central to processing rewarding and aversive stimuli^40^, and is critical in mediating depression symptomatology^41,42^. Confocal microscopy revealed that brain-infiltrating monocytes were attached to the vasculature in NAc but did not infiltrate the brain parenchyma (Fig. 2e).

**Extended Data Fig. 5.**
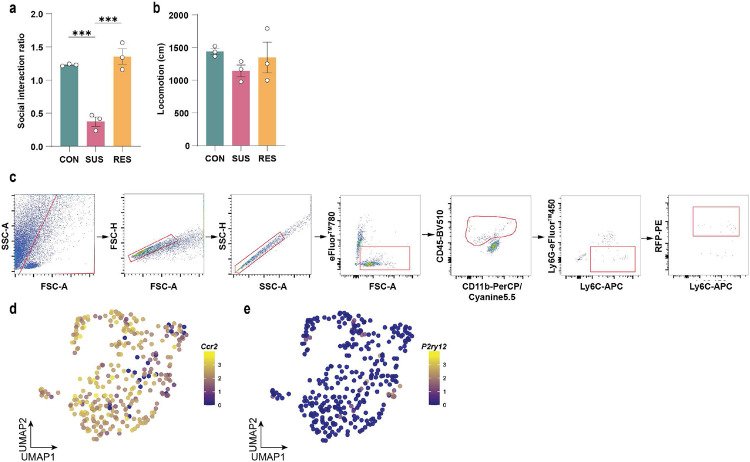
Single-cell RNA-sequencing of brain-infiltrating *Ccr2*^rfp+^ monocytes. **a,** Social interaction ratio and **b,** locomotion of unstressed control (CON), susceptible (SUS) and resilient (RES) mice (One-way ANOVA with Bonferroni *post hoc* test). **c,** Gating strategy for fluorescence-activated cell sorting of brain-infiltrating monocytes. Feature plots of the **d,** monocyte enriched gene *Ccr2* and **e,** the microglia enriched gene *P2ry12*. *** p < 0.001. Data are shown as means ± s.e.m.

**Extended Data Fig. 6.**
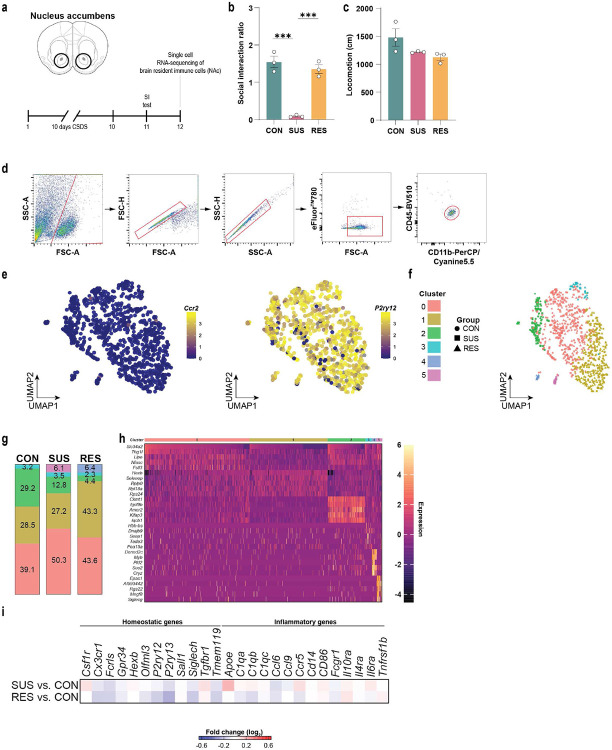
Single-cell RNA-sequencing of brain-resident myeloid cells in the nucleus accumbens (NAc). **a,** Experimental outline. **b,** Social interaction ratio and **c,** locomotion of unstressed control (CON), susceptible (SUS) and resilient (RES) mice (One-way ANOVA with Bonferroni *post hoc* test). **d,** Gating strategy for fluorescence-activated cell sorting of brain-resident myeloid cells. **e,** Feature plots. **f,** Uniform Manifold Approximation and Projection (UMAP) representation of 6 clusters identified using Seurat clustering. **g,** Relative (%) abundance of number of cells per cluster in CON, SUS and RES mice. Cluster 5 was specific to SUS mice and cluster 4 was specific to RES mice, however, gene ontology analyses did not reveal any significantly enriched pathways. **h,** Heat map of top five cluster defining protein coding genes. **i,** heatmap of homeostatic and inflammatory genes. There were no significant differences (p-value adjusted < 0.05) in these homeostatic or inflammatory genes. *** p < 0.001. Data are shown as means ± s.e.m.

Next, we investigated how brain-infiltrating monocytes contribute to stress-induced social avoidance and performed single-cell RNA-sequencing of brain-infiltrating monocytes after CSDS (Fig. 2a, Extended Data Figs. 5a-e). Unsupervised clustering of *Ccr2*^rfp+^ monocytes revealed four unique clusters based on their transcriptional profiles (Fig. 2f). We found cluster 0 to be enriched in SUS mice relative to CON and RES mice (Fig. 2g). To determine cluster-defining genes, we performed a differential gene expression analysis by investigating differentially expressed genes between clusters and total genes (adjusted p-value < 0.05) (Fig. 2h). Several genes known to be involved in inflammatory processes were found to be upregulated in Cluster 0: e.g., genes coding for S100 proteins, including S100A6 and S100A11^43,44^, *Lyz1* coding for lysozyme, an antimicrobial protein critical for host defense^45^, and the annexins *Anxa1* and *Anxa2*^46^. GO term analysis of upregulated genes of cluster 0 revealed involvement in oxidation-reduction process (GO:0055114), extracellular space (GO:00056150) and extracellular matrix (GO:003102) (Fig. 2i). One of the top genes enriched in these extracellular space/matrix pathways was Mmp8, which codes for matrix metalloproteinase 8 (Figs. 2j, k). Of note, *Mmp8* was also one of the top differentially expressed genes in circulating in Ly6C^high^ monocytes from SUS vs. CON mice, as was the GO term extracellular space (Figs. 1l, m). We also performed single-cell RNA-sequencing of brain-resident immune cells in NAc (Extended Data Figs. 6a-e), however, we did not observe any stress-induced changes in homeostatic or inflammatory gene signatures (Extended Data Figs. 6f-i). While previous studies in mice have reported stress-induced morphological, transcriptional and functional changes indicative of an inflammatory signature in microglia from other brain regions^47,48,49,50^, this is the first study investigating cell type-specific gene expression signatures at the single-cell level in NAc microglia. This finding suggests that there are brain-region-specific differences in microglial reactivity to stress, which is in line with recent studies showing substantial heterogeneity in microglia across brain regions^51^. Our results are also consistent with recent single-cell studies conducted in postmortem brain samples from human subjects with MDD, where no evidence of pro-inflammatory microglia signatures was found^52,53^.

MMP8 belongs to the group of collagenases and is derived and secreted largely from neutrophils and monocytes^21,54,55^. Our data and published studies suggest that, unlike many other MMPs, MMP8 is not produced or secreted by any cells of the central nervous system, including brain-resident myeloid cells^54^ (Fig. 2l). Interestingly, in one of the largest whole blood gene expression studies to date, MMP8 was one of the top upregulated genes in patients with MDD compared to HC^56^. In addition, Song et al. reported a single-nucleotide polymorphism in the coding region of *MMP8* to be associated with MDD^57^. However, the underlying mechanisms linking MMP8 with MDD have not been explored. Activated MMP8 can cleave a wide range of ECM components, such as collagens, fibronectins, tenascins and aggrecan, many of which are components of the brain ECM^20,54^.

## Peripheral MMP8 is associated with brain extracellular space abnormalities

**Extended Data Fig. 7.**
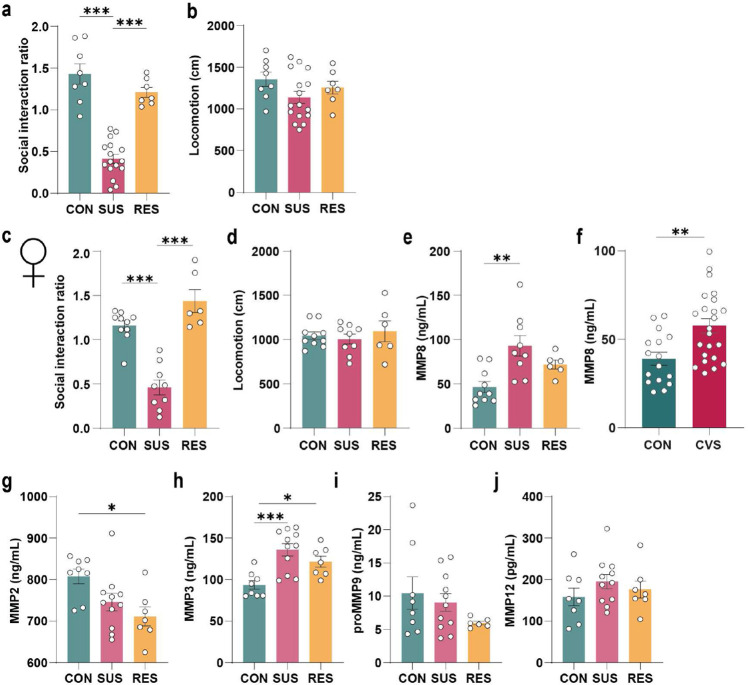
Stress increases matrix metalloproteinase 8 (MMP8) in both male and female mice. **a,** Social interaction ratio and **b,** locomotion of unstressed control (CON), susceptible (SUS) and resilient (RES) male mice (One-way ANOVA with Bonferroni *post hoc* test). Defeat characteristics of female mice after 10 days of chronic social defeat stress (CSDS). (**c,** social interaction ratio, **d,** locomotion) (One-way ANOVA with Bonferroni *post hoc* test). **e,** Plasma levels of MMP8 in SUS compared to CON female mice after 10 days of CSDS. (One-way ANOVA with Bonferroni *post hoc* test). **f,** MMP8 plasma levels of female mice that underwent a 21-day paradigm of chronic variable stress (CVS) compared to non-stressed control mice (two-tailed Student’s t-test). Plasma levels of **g,** MMP2, **h,** MMP3, **i,** proMMP9 and **j,** MMP12 in CON, SUS and RES male mice after 10 days of CSDS. * p < 0.01, ** p < 0.01, *** p < 0.001. Data are shown as means ± s.e.m.

**Extended Data Fig. 8.**
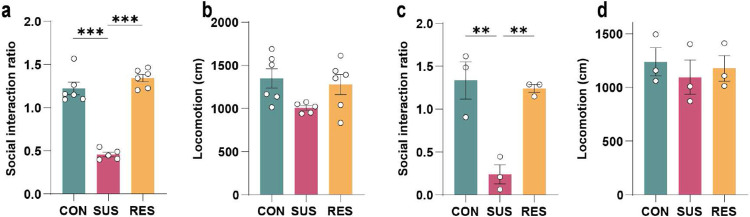
Defeat characteristics of mice assessed for brain matrix metalloproteinase 8 (MMP8) and alterations in brain extracellular space. **a,** Social interaction ratio and **b,** locomotion of unstressed control (CON), susceptible (SUS) and resilient (RES) mice from which levels of MMP8 in the nucleus accumbens (NAc) were analysed (One-way ANOVA with Bonferroni *post hoc* test, each data point represents three pooled mouse brains). **c,** Social interaction ratio and **d,** locomotion of CON, SUS and RES mice analysed for the transmission electron microscopy experiment. (One-way ANOVA with Bonferroni *post hoc* test). ** p < 0.01, *** p < 0.001. Data are shown as means ± s.e.m.

**Fig. 3.**
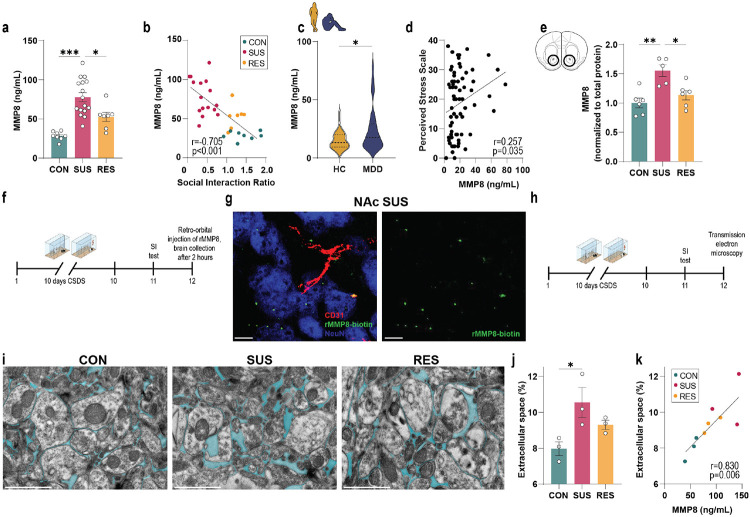
Stress-induced increase in matrix metalloproteinase 8 (MMP8) is associated with altered extracellular space in the nucleus accumbens (NAc). **a,** Plasma levels of MMP8 in unstressed control (CON), susceptible (SUS) and resilient (RES) mice (One-way ANOVA with Bonferroni *post hoc* test). **b,** Correlation of MMP8 in circulation with social interaction ratio (Pearson correlation). **c,** Serum MMP8 levels in patients with major depressive disorder (MDD) and healthy controls (HC) (two-tailed Student’s t-test). **d,** Correlation between MMP8 and Perceived Stress Scale (Pearson correlation). **e,** Normalized MMP8 protein levels in NAc lysates of CON, SUS and RES mice. (One-way ANOVA with Bonferroni *post hoc* test, each datapoint represents three pooled NAc). **f,** Experimental outline of recombinant (r)MMP8 biotinylation. **g,** Representative immunofluorescence image of the NAc of SUS mice that were injected retro-orbitally with biotinylated (r)MMP8. Scale bar: 10 μm. **h,** Outline of transmission electron microscopy experiment to assess the extracellular space in NAc. **i,** representative TEM images of CON, SUS and RES mice. Scale bar: 1 μm. **j,** Quantification of the extracellular space relative to total brain area (One-way ANOVA with Bonferroni *post hoc* test). **k,** Correlation between plasma MMP8 levels and extracellular space volume fraction (Pearson correlation). * p < 0.05, ** p < 0.01, *** p < 0.001. Data are shown as means ± s.e.m.

To test whether monocyte-derived MMP8 can promote stress susceptibility, we first confirmed the stress-induced increase in MMP8 at the protein level in plasma after CSDS (Fig. 3a, Extended Data Figs. 7a, b), and showed that MMP8 levels were negatively correlated with SI ratio (Fig. 3b). We further demonstrated that both 10 days of CSDS and 21 days of chronic variable stress increased plasma levels of MMP8 in female mice (Extended Data Figs. 7c-f). Next, we measured plasma levels of other MMPs such as MMP2, 3, 9 and 12 in the same mice as shown in Fig. 3a after 10 days of CSDS. While we did observe a modest increase of MMP3, we observed similar changes in both SUS and RES mice compared to CON mice, confirming that only MMP8 is uniquely upregulated in SUS mice but not CON or RES mice (Extended Data Figs. 7g-j). Finally, we validated the increased MMP8 in serum from patients with MDD compared to HC (Fig. 3c, Supplementary Table 4) and found a positive correlation with self-reported perceived stress (Fig. 3d). We then confirmed that MMP8 was increased in the NAc of SUS mice following CSDS (Fig. 3e, Extended Data Figs. 8a, b). By retro-orbitally injecting biotinylated mouse recombinant MMP8 (rMMP8) into stress-susceptible mice (Fig. 3f), we showed that peripheral MMP8 can access the brain parenchyma (Fig. 3g).

We then investigated whether CSDS affects the brain extracellular space using transmission electron microscopy imaging of NAc tissue sections (Fig. 3h). SUS mice showed increased extracellular space volume fractions in the NAc compared to CON mice (Figs. 3i, j, Extended Data Figs. 8c, d). Increases in brain extracellular space volume have been described in other central nervous system pathologies, such as neurodegenerative disorders, and can be a result of disrupted cell-attachments associated with degradation of the ECM^58^. Notably, extracellular space volume fractions in the NAc positively correlated with peripheral MMP8 in the same mice (Fig. 3k).

## MMP8 depletion attenuates stress-induced social avoidance behaviour

**Extended Data Fig. 9.**
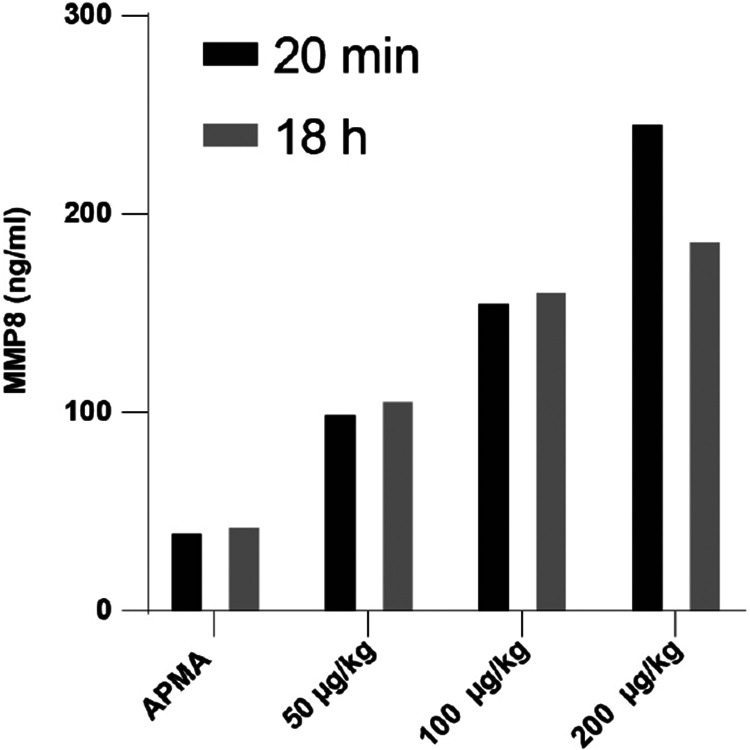
Dose response study for intra-peritoneal (i.p.) injection of recombinant matrix metalloproteinase 8 (rMMP8). Different doses of rMMP8 or vehicle (4-aminophenylmercuric acetate, APMA) were injected i.p. and blood was collected 20 min or 18 h after the injection followed by MMP8 measurement in plasma.

**Fig. 4.**
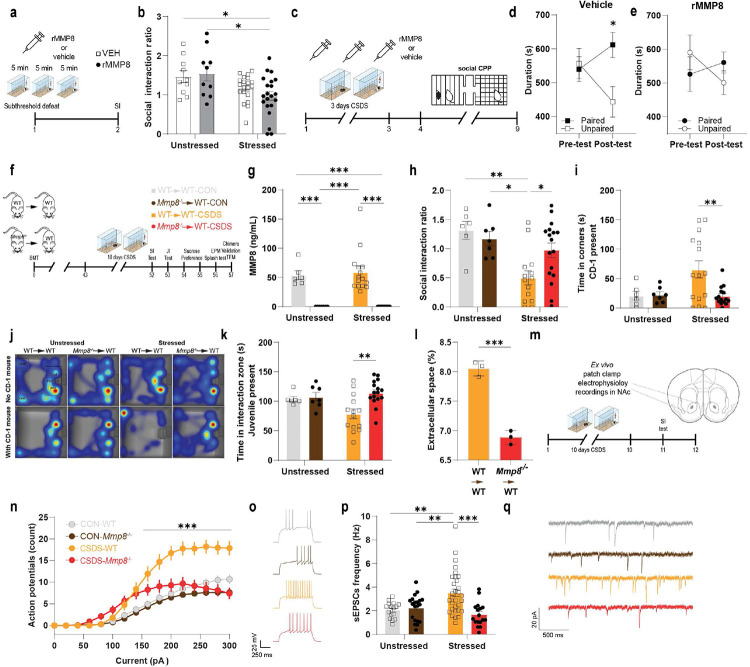
Peripheral matrix metalloproteinase 8 (MMP8) is causally linked to stress-induced social avoidance behaviour, alterations in brain extracellular space and neurophysiology. **a,** Experimental outline of mice receiving either 100 μg/kg recombinant (r)MMP8 or vehicle (VEH) followed by a subthreshold stress. **b,** Social interaction (SI) ratio of mice injected with rMMP8 followed by a subthreshold defeat. **c,** Outline of social conditioned place preference (CPP) experiment. **d,** VEH injected mice show CPP towards a chamber that was paired with a juvenile mouse, **e,** while this was abolished in mice injected with rMMP8 during a subthreshold defeat. **f,** Outline of bone marrow chimera experiment, with bone marrow transplantation (BMT) on day 0, followed by 6 weeks of recovery and then chronic social defeat stress (CSDS). After CSDS, mice were tested in a comprehensive behaviour battery, consisting of a SI test with a novel CD-1 mouse, a juvenile interaction (JI) test with a novel same-sex juvenile mouse, sucrose preference test, elevated plus maze (EPM) and splash test. On the day of sacrifice, chimerism was validated and brains of a subset of stressed mice were processed for transmission electron microscopy (TEM) imaging of the extracellular space. **g,** Plasma levels of MMP8. **h,** SI ratio and **i,** time spent in the corner as a measure of social avoidance. **j,** Representative heat maps of behaviour during social interaction test. **k,** Time spent in the interaction zone during a JI test when the novel juvenile mouse was present. **l,** Quantification of the extracellular space relative to total brain area in WT compared to *Mmp8*^−/−^ chimeras. **m,** Outline of *ex vivo* patch clamp experiment in nucleus accumbens (NAc) medium spiny neurons. **n,** Action potentials in *Mmp8*^−/−^ and WT mice after 10 days of CSDS. **o,** Representative traces of action potentials evoked upon a +120 pA step of depolarizing current. **p,** Frequencies of spontaneous excitatory postsynaptic currents (sEPSCs) in *Mmp8*^−/−^ and WT mice after 10 days of CSDS. **q,** Representative traces of sEPSCs. (Two-way ANOVAs followed by Tukey’s *post hoc* testing (for panels b, d, g, h, i, k, n, p) or two-sided Student’s t-test (for panel l). * p < 0.05, ** p < 0.01, *** p < 0.001. Data are shown as means ± s.e.m.

Next, we determined whether MMP8 is causally linked to stress-induced social avoidance. First, we tested if the combination of an intraperitoneal injection of rMMP8 (at a dose that leads to similar plasma levels of MMP8 as observed in SUS mice after CSDS [Extended Data Fig. 9]) coupled with subthreshold defeat – a shorter version of CSDS that in wildtype mice does not lead to behavioural alterations^40^ – promotes stress susceptibility (Fig. 4a). Indeed, we found that the combination of rMMP8 and subthreshold stress led to a lower SI ratio compared to unstressed mice (Fig. 4b). We then tested if rMMP8 also changed social preference by testing mice with a non-threatening same-sex juvenile mouse using a social conditioned place preference (sCPP) test (Fig. 4c). This paradigm has been used historically to assess social reward^59^. While mice that received vehicle injections during three days of a subthreshold CSDS did form a preference for the chamber that was previously paired with the juvenile mouse (Fig. 4d), the social preference was abolished in mice that received rMMP8 (Fig. 4e).

**Extended Data Fig. 10.**
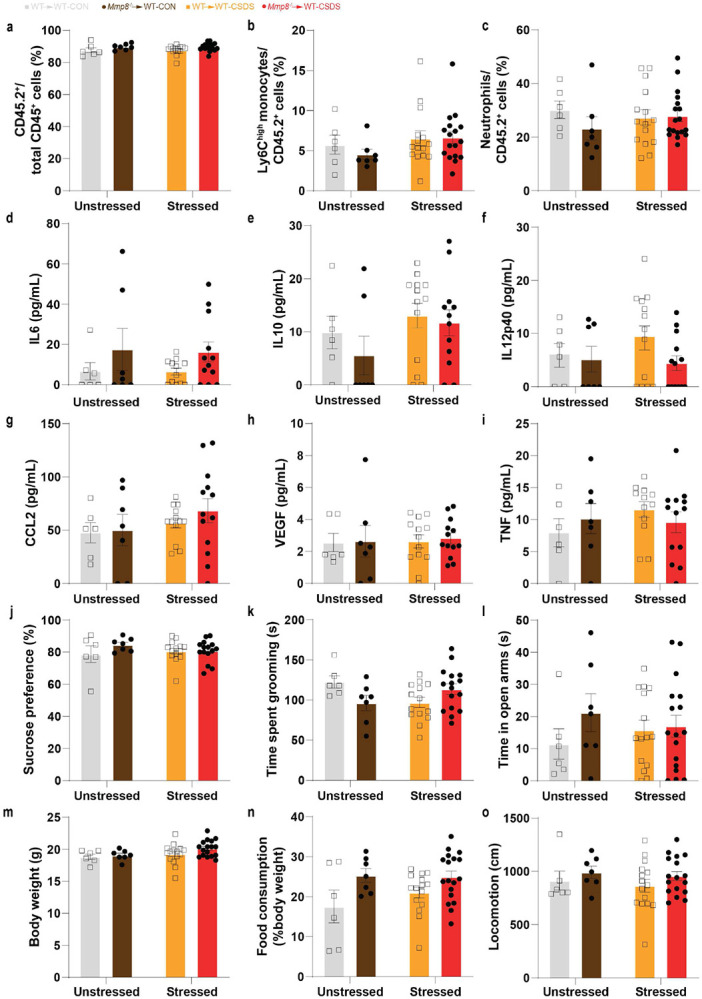
Bone marrow chimera experiment. **a,** Chimerism (CD45.2^+^ vs. total CD45^+^ cells) of unstressed and stressed WT and *Mmp8*^−/−^ transplants. Frequencies of circulating **b,** Ly6C^high^ monocytes and **c,** neutrophils. Plasma levels of **d,** Interleukin 6 (IL6), **e,** IL10, **f,** IL12p40, **g,** C-C motif chemokine ligand 2 (CCL2), **h,** Vascular endothelial growth factor (VEGF) and **i,** Tumor necrosis factor (TNF). **j,** Sucrose preference test, **k,** splash test and **l,** elevated plus maze test. Sickness associated behaviours such as **m,** body weight, **n,** food consumption or **o,** locomotion. (Two-way ANOVA followed by Tukey’s *post hoc* testing [for all panels]). Data are shown as means ± s.e.m.

To selectively deplete *Mmp8* in peripheral leukocytes, we created chimeric mice that lack *Mmp8* specifically in peripheral leukocytes (*Mmp8*^−/−^→WT) or wild-type controls (WT→WT) by bone marrow transplantation (BMT) with hematopoietic stem cells from *Mmp8*^−/−^ or *Mmp8*^+/+^ (WT) donor mice. These chimeric mice were then exposed to CSDS and underwent behavioural testing and assessment of the extracellular space in NAc (Fig. 4f). We first validated the efficiency of the BMT experiment and found a complete depletion of MMP8 in the blood of *Mmp8*^−/−^→WT mice not only validating the efficiency of the BMT but confirming that the source of MMP8 is indeed from peripheral leukocytes (Fig. 4g). We also found high chimerism (~85-90%) and no differences in frequencies of peripheral monocytes, neutrophils, cytokines or chemokines between the wild-type and knockout BMT mice (Extended Data Figs. 10a-i), suggesting that peripheral depletion of MMP8 does lead to major changes in the peripheral immune system. Behaviourally, mice that were transplanted with hematopoietic stem cells from *Mmp8*^−/−^ mice showed less social avoidance from a CD-1 mouse compared to stressed WT→WT mice, as measured by social interaction ratio and time the experimental mouse spent in the corner (Figs. 4h-j). Similar effects were observed when mice were tested for SI with a same-sex juvenile mouse (Fig. 4k). Interestingly, we did not observe any effects of MMP8 depletion on changes in other non-social stress-related behaviours such as sucrose preference test, splash test or elevated plus maze (Extended Data Figs. 10j-l). We also did not observe differences in sickness-related behaviours such as body weight, food consumption or general locomotion (Extended Data Figs. 10m-o).

## MMP8 depletion regulates brain extracellular space and neurophysiology

**Extended Data Fig. 11.**
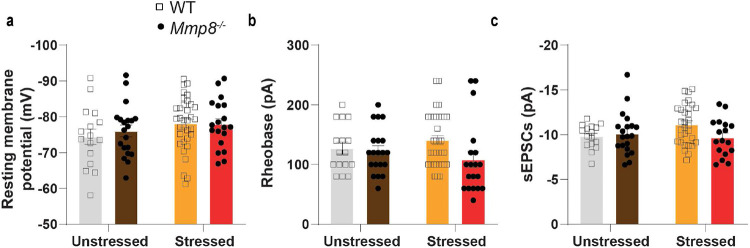
*Ex vivo* patch clamp electrophysiology in nucleus accumbens medium spiny neurons. **a,** Resting membrane potential, **b,** rheobase and **c,** amplitude of spontaneous excitatory postsynaptic potentials (sEPSCs). (Two-way ANOVA followed by Tukey’s *post hoc* testing [for all panels]). Data are shown as means ± s.e.m.

Lastly, we sought to assess whether MMP8 is linked to changes in extracellular space and NAc neurophysiology. Again, using transmission electron microscopy we assessed the volume of extracellular space in stressed mice and found that *Mmp8*^−/−^→WT mice had reduced extracellular space compared to WT→WT mice (Fig. 4l). Given that ECM changes in general and MMP-mediated reorganization of the ECM in particular have been previously associated with altered neuronal physiology^24,55,60,61^, we performed ex vivo whole cell patch clamp recordings of medium spiny neurons (MSNs) from the NAc of *Mmp8*^−/−^ and WT mice (Fig. 4m). Previous studies have shown that CSDS leads to neurophysiological changes in NAc MSNs of SUS but not RES mice, including increased intrinsic neuronal excitability and increased frequency of excitatory postsynaptic currents (EPSCs)^62,63^. In line with our hypothesis, a lack of *Mmp8* attenuated stress-induced increased neuronal excitability (Figs. 4n, o) and spontaneous EPSCs (Figs. 4p, q), without affecting the resting membrane potential, rheobase or the amplitude of spontaneous EPSCs (Extended Data Figs. 11 a-c).

**Extended Data Fig. 12.**
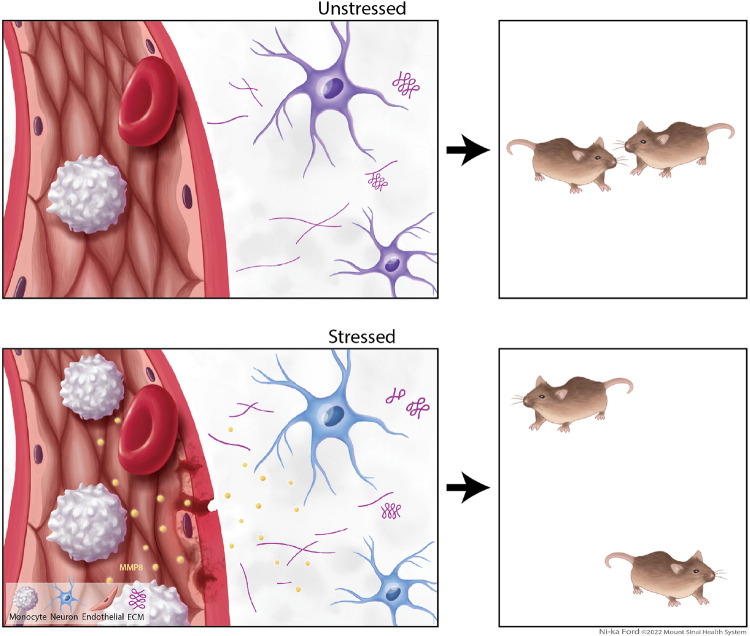
Graphical abstract. Stress-induced increase in peripheral MMP8 leads to social avoidance behavior associated with alterations in the extracellular space (ECM) and neurophysiological changes in the nucleus accumbens.

In the current study, we have shown that a stress-induced increase in peripheral MMP8 leads to alterations in the extracellular space of the NAc associated with altered NAc neurophysiology and social avoidance (Extended Data Fig. 12). This study provides evidence for a novel mechanism by which the peripheral immune system can affect neuronal function and behaviour. While several studies have causally linked peripheral immune factors like cytokines or different cell types to behavioural alterations, they have shown or hypothesized mechanisms affecting neurons directly, e.g., by binding of cytokines to receptors expressed on neurons^14,64,65,66^. Here, we demonstrate a novel way in which stress promotes peripheral immune cell interactions with the brain to control behaviour—i.e., immune cell-derived MMPs from the circulation that affect neuronal function via potentially changing the extracellular space. While future studies are needed to better define the ECM substrates of MMP8 involved, and to identify additional peripheral and central factors affecting ECM homeostasis, these data provide important insights into the emerging role that neuroimmune mechanisms have in neuropsychiatric disorders, highlighting new peripheral targets for advancing biomarker and treatment options.

## Materials and methods

### Mice.

The following mouse strains were used: For standard chronic social defeat stress (CSDS) experiments, 7 week-old C57BL/6J (Stock#: 000664) mice were purchased from The Jackson Laboratory. For bone marrow transplantation experiments, 4 week-old B6.SJL-*Ptprc^a^ Pepc^b^*/BoyJ (Stock#: 002014, B6 CD45.1) mice were ordered from The Jackson Laboratory. B6.129(Cg)-*Ccr2^tm2.1Ifc^*/J (STOCK#: 017586, *Ccr2*^rfp^) and B6.129X1-*Mmp8*^tm1Otin^/J (Stock#: 005514, *Mmp8*^−/−^), were bred inhouse. 4-6 month-old male retired CD-1 breeders (Charles River Laboratories, Crl:CD1[ICR]) were used as aggressors for male CSDS. For the female CSDS experiment, male B6N.129S6(Cg)-*Esr1^tm1.1(cre)And^*/J (Stock#: 017911, *ERα*-Cre) mice were purchased from The Jackson Laboratory and were crossed with CD-1 females to obtain F1 males, which were used as aggressors. Mice purchased from external vendors were allowed to habituate to the animal facility for at least 1 week. Animals were maintained on a 12 h light/dark cycle (lights on at 7 am, lights off at 7 pm) with *ad libitum* access to food and water. For all behavioural tests, mice were allowed to acclimate to the testing room for at least 1 h. All procedures were performed in accordance with the National Institutes of Health Guide for Care and Use of Laboratory Animals and the Icahn School of Medicine at Mount Sinai (ISMMS) Institutional Animal Care and Use Committee.

### CSDS and social interaction (SI) test.

For male CSDS^27^, retired male CD-1 breeders (age: 4-6 months) were used as aggressors. Before each defeat, aggressors were screened for aggressive behaviour for three consecutive days based on previously described criteria^27^. Two days before the start of the defeat, CD-1 mice were housed on one side of a perforated Plexiglas partition. During 10 consecutive days of CSDS, experimental mice (7–8 week-old) were subjected to direct physical interaction with a CD-1 for 10 min per day (5 min for bone marrow chimera cohorts), and the rest of the day placed on the other side of the Plexiglas divider, allowing for sensory but not direct physical contact. Male aggressors for female CSDS^67,68^ were generated as follows: Heterozygous ERα-Cre mice were bilaterally injected with a Cre-dependent AAV-DIO-hM3D(Gq)-DREADD (Addgene, 44361-AAV2) into the ventrolateral subdivision of the ventromedial hypothalamus. To activate ERα^+^ cells, intraperitoneal (i.p.) injections of 1.0 mg/kg clozapine-N-oxide (Tocris, #4936) were administered 30 min before each defeat bout. Unstressed control mice were pair-housed across a Plexiglas partition. After the last day of defeat, stressed and unstressed control mice were singly housed (males) or kept in pairs (females). All stressed mice were carefully examined for wounding during the CSDS experiments and mice with excessive wounding were excluded.

### Subthreshold stress.

Subthreshold stress is a variation of the CSDS paradigm that is used to unravel pro-susceptible factors^40^ without eliciting behavioural alterations in unmanipulated mice. Experimental mice were exposed to three 5 min periods of direct physical interactions with an aggressive CD-1 mouse with a 15 min interval between defeats. 24 h after the last defeat bout, SI test was conducted as described below.

### Social interaction (SI) test.

SI test was performed 24 h after the last defeat session under red light conditions. After a 1 h habituation period to the behavioural suite, mice were placed into a Plexiglas arena (42 cm × 42 cm × 42 cm, Nationwide Plastics) with a small meshed enclosure on one end. For the first 2.5 min, the experimental mouse freely explored the arena. The mouse was then removed from the arena which was subsequently cleaned with 70 % ethanol, then, a novel social target (CD-1 for males and *ERα*-Cre for female CSDS) was placed into the enclosure and the experimental mouse was placed back into the arena for another 2.5 min. Locomotor activity was tracked and recorded using a Noldus Ethovision System (Noldus Information Technology Inc, Version 11.0, Leesburg, VA). SI ratio was calculated as the ratio between the time the experimental mouse spent in the vicinity of the enclosure (SI zone) when a target mouse was present vs. absent. Mice with an SI ratio of ≥ 1 show a behavioural profile similar to unstressed control mice and were termed resilient, while mice with an SI ratio < 1 were termed susceptible. To test social avoidance behaviour towards a juvenile mouse, SI test was performed as described above with a 4–6 week-old male juvenile mouse as a social target. Additional parameters that were measured were total locomotion and time spent in corners, calculated as the sum between the two corners opposite the wire enclosure.

### Chronic variable stress (CVS).

CVS was conducted in female mice as previously described^69^. For 21 days, animals were exposed to daily 1 h long stressors, consisting of either 100 mild foot shocks (0.45 mA), restraint stress in a 50 mL falcon tube, or tail suspension. For the duration of the stress, mice were group housed.

### I.p. injection of recombinant matrix metalloproteinase 8 (rMMP8).

Before injection, rMMP8 (Bio-techne, #2904-MP-010) was activated ex vivo for 1 h at 37 °C with 1 mM 4-aminophenylmercuric acetate (APMA) in mercury-containing assay buffer (Anaspec, #AS-71154) and then diluted in 0.9 % sterile saline solution (VWR, #101448-952). For the dose-response experiment, we injected 3 three different doses 50, 100 and 200 μg/kg, and blood was drawn 20 min after the injection via submandibular bleeding and 18 h post-injection using trunk blood. MMP8 was measured as described below. For the behavioural experiments, mice were injected with 100 μg/kg rMMP8 or APMA 20 min before the defeat bout.

### Generation of bone marrow (BM) chimeras.

BM chimeras were generated as described before^14,34^. To ablate the peripheral immune system of the host mouse, 5- week-old male B6 CD45.1 mice were irradiated with a total of 11 Gy, delivered in two doses of 5.5 Gy, 3-4 h apart (X-rad 320 Irradiator [Precision X-Ray, Madison, CT]). Hematopoietic progenitor cells were isolated from the femur/tibia of either *Mmp8*^−/−^ or *Mmp8*^+/+^ male donor mice (12 weeks old). 1 h after the second dose of irradiation, 1 x 10^6^ cells were injected retro-orbitally in mice anaesthetized with isoflurane. Host mice were then allowed to recover for a total of six weeks. Mice received antibiotic treatment (0.2 % in drinking water) (Neomycin trisulfate, N1876, Sigma) during the first three weeks of recovery. Level of chimerism was assessed using flow cytometry (comparing CD45.1 (host) (mouse anti-CD45.1-PE-Cyanine7, clone A20, Invitrogen, #25-0453-81) and CD45.2 (donor) (mouse anti-CD45.2-BV421, clone 104, BD Bioscience, #562895) leukocytes, and measuring MMP8 in plasma (Abcam, #ab206982).

### Social conditioned place preference (sCPP).

sCPP was performed as previously described^70^. The experiment was done under red light conditions after mice were habituated to the CPP room for 1 h. The CPP chamber (Med Associates) consisted of 3 different compartments: a neutral middle part, and two adjacent chambers, each with distinct floors (grid pattern) and walls. On the pre-test day, mice were allowed to explore all three chambers for 20 min and the time spent in each chamber was recorded. Based on these durations, mice were balanced to account for pre-test preferences. During the four consecutive conditioning days, mice were conditioned twice per day: In the morning, mice were placed in one chamber for 15 min with a novel, same-sex juvenile (4-5 week-old) C57BL/6J mouse (paired chamber). In the afternoon, the experimental mouse was put in the empty opposite chamber for the same amount of time (unpaired chamber). On the testing day, mice were again allowed to freely explore all chambers for 20 min and the time spent in each chamber was automatically recorded (Med Associates).

### Sucrose preference test.

Sucrose preference test was performed to assess hedonic behaviour towards a sweet gustatory stimulus^16^. Mice were given access to two water bottles (50 mL conical tubes with sipper tops) for 24 h for habituation. Then, one water bottle was exchanged with a bottle containing 1% sucrose (Sigma, #S0389) in drinking water. After 24 h, the bottle positions were swapped to prevent position bias. After another 24 h, sucrose preference was assessed as follows (based on weight of bottles): [sucrose (g) / total fluid (g)] x 100.

### Splash test.

The splash test, a test performed to assess self-care behaviour, was conducted under red-light conditions as described previously^16^. Briefly, after 1 h of habituation to the testing room, a 10% sucrose solution was gently sprayed onto the lower back of the mouse. Behaviour was recorded for 5 min, and time spent grooming was scored.

### Elevated plus maze test (EPM).

The EPM was conducted to assess anxiety-like behaviours^16^. After 1 h of habituation to the testing room, mice were placed on an elevated cross-shaped maze for 5 min under red light conditions. The four arms (two arms without and two arms with walls, each arm of the maze measuring 12 x 50 cm) were elevated 1 m above the floor. Behaviour was tracked using a Noldus Ethovision System (Noldus Information Technology Inc, Version 11.0, Leesburg, VA). Parameters assessed included time spent in closed arms, open arms and in the center.

### Mass cytometry (CyTOF).

Blood was collected directly into fluorescence-activated cell sorting (FACS) buffer (DPBS (Thermo Fisher Scientific, #14190144) containing 0.5% bovine serum albumin (Sigma Aldrich, #A9647) and 2 mM EDTA (Invitrogen, #AM9260G). Cells were pelleted and red blood cells (RBCs) were lysed using RBC lysis buffers (BD, #555899). Immune cells of the brain were isolated as previously described^31^. Briefly, mice were anaesthetized with 10% chloral hydrate and transcardially perfused with ice-cold PBS (0.1 M). Brains were then cut into small pieces using scissors in a total of 3 mL digestion buffer (RPMI (Thermo Fisher Scientific, #11875093) with 2% fetal bovine serum (Thermo Fisher Scientific, #A3840001), 2 mM HEPES (Corning, #25-060-CI) and 0.4 mg/mL collagenase D (Roche, #12352200). The cell suspension was then incubated for 30 min at 37 °C. Digestion was stopped by adding EDTA (Invitrogen, #AM9260G) to a final concentration of 5 mM. Using blunt 18 G needles (BD, #303129), the cell suspension was gently homogenized, and the homogenate was passed through a 70 μm strainer (pre-wet with PBS) (Miltenyi Biotec, #130-095-823). Cells were pelleted, resuspended in 30% Percoll (Millipore Sigma, GE17-0891-01) and centrifuged for 30 min at 23,500 g without brakes at 4 °C. The myelin layer was aspirated and the middle layer containing leukocytes was transferred into a conical tube. Cells were then washed and stained for 30 min on ice with a mix of metal-conjugated antibodies (**Supplementary Table 1**). After antibody staining, cells were incubated with cisplatin for 5 min at room temperature as a viability dye to enable exclusion of dead cells. Cells were then fixed in PBS containing 1.6% formaldehyde and a 1:4,000 dilution of Ir nucleic acid intercalator to label all nucleated cells. Immediately prior to acquisition, cells were washed in PBS, then in distilled water, and finally resuspended in distilled water containing a 1/10 dilution of Equation 4 Element Calibration beads (Fluidigm, #SKU 201078). After routine instrument tuning and optimization, the samples were acquired on a CyTOF2 Mass Cytometer equipped with a Super Sampler fluidics system (Victorian Airships). The acquisition rate was < 500 events/s. The resulting FCS files were concatenated and normalized using a bead-based normalization algorithm in the CyTOF acquisition software and uploaded to Cytobank (https://mtsinai.cytobank.org/cytobank/; Cytobank, Menlo Park, CA, 7.0). FCS files were manually pre-gated for CD45^+^ events, excluding dead cells, doublets and DNA-negative debris (Extended Data Fig. 1a). Data analysis was performed with Clustergrammer, a web-based tool for visualizing and analysing high-dimensional data (https://github.com/ismms-himc/LegendScreen_CyTOF).

### FAC-sorting and bulk-RNA sequencing of leukocyte subpopulations.

For the mouse leukocyte subpopulation sequencing experiment, trunk blood was collected directly into FACS buffer. Samples were centrifuged and RBC lysis was performed (BD, #555899). After washing the cell pellet with ice-cold DPBS, Fc receptor blocking (rat anti-CD16/CD32, clone 2.4G2, BD Biosciences, #553141) was performed on ice for 30 min. Cells were pelleted and washed once. Leukocytes were then stained with the following antibodies (all at 1:400): rat anti-CD11b-PE-Cyanine7 (clone M1/70, BioLegend, #101215), rat anti-Ly6C-PerCP-Cy5.5 (clone HK1.4, BioLegend, #128027), rat anti-Ly6G-PE (clone 1A8, BioLegend, #127607), rat anti-B220-FITC (clone RA3-6B2, BioLegend, #103205) and rat anti-CD90.2-APC (clone 53-2.1, BioLegend, #140312) for 30 min on ice protected from light. After an additional wash, cells were sorted directly into Trizol (Themo Fisher Scientific, #15596026) by a BD FACSAria II cell sorter. Raw flow cytometry data were analysed using FlowJo software (FlowJo LLC, Version 10.6.2). Samples were flash frozen on dry ice and stored at −80 °C. RNA was extracted using the RNeasy Micro Kit according to the manufacturer’s instructions (Qiagen, #74004). RNA quality, RNA integrity number (RIN) and RNA concentrations were assessed using Nanodrop (Thermo Fischer Scientific) and Bionalyzer (Agilent, #5067-1513). 500 pg of purified RNA was used for library preparation, which was performed using the SMARTer Stranded Total RNA-Seq Kit v2 - Pico Input Mammalian (Takara, #634413). Libraries were barcoded for multiplexing. Before sequencing, library quality and concentration were measured using Qubit Fluorometric Quantitation (Thermo Fisher). Libraries were sequenced (2 x 150 base pair, paired-end reads configuration, v4 chemistry) on an Illumina HiSeq machine at a minimum of 30 million reads per sample. Sequencing was performed at Genewiz. Raw sequencing reads from the samples were mapped to mm10 using HISAT2 v2.1.0^71^. Counts of reads mapping to genes were obtained using htseq-count v0.12.4 against Ensembl v90 annotation^72^. Differential expression analysis was done using DESeq2 v1.26.0 package^73^. The fold change threshold was set at 2 (i.e., log2 fold change > ∣1∣). Gene ontology terms were determined using the Database for Annotation, Visualization, and Integrated Discovery (DAVID), version 6.8^74,75^. Only gene ontology terms with an adjusted p-value < 0.05 (FDR) and an overall of > 5% (involved genes/total genes) were considered.

### FACS and single-cell RNA-sequencing.

Brain-infiltrating monocytes and microglia were isolated based on previous published protocols^76^. 24 h after the SI test, mice were euthanized by injecting 10% chloral hydrate and perfused transcardially with ice-cold 0.1 M PBS (pH 7.4). Brains were rapidly dissected and put in ice-cold PBS (for brain-infiltrating monocyte RNA-sequencing experiment) or bilateral NAc tissue punches were obtained from 1 mm thick coronal slices using 1.2 mm punches (for resident myeloid cell RNA-sequencing experiment) (GE Healthcare Life Sciences, #1205X41). All the following steps were performed strictly on ice. For whole brains, tissue was cut into small pieces, for punches no shredding was needed. Tissue was then transferred to DPBS and homogenized with pestles (Sigma, #D8938-1) in ice-cold PBS (20 stokes with pestle A, 20 stokes with pestle B). The cell suspension was then passed through a 70 μm cell strainer (pre-wet with PBS) (Miltenyi Biotec, #130-095-823) into a 15 mL conical tube. Cells were pelleted (300 g for 5 min at 4 °C), resuspended in 10 mL of ice-cold 40% isotonic Percoll (Millipore Sigma, GE17-0891-01) (diluted in PBS) and centrifuged for 30 min at 500 g at 4 °C with full acceleration and braking. The myelin layer was aspirated, then the cell pellet was washed with 10 mL of ice-cold PBS by centrifuging at 300 g for 5 min at 4 °C. Cells were then resuspended in FACS buffer, Fc receptor binding was blocked (rat anti-CD16/CD32, clone 2.4G2, BD Biosciences, #553141) and then cells were stained with a viability dye (Thermo Fisher Scientific, #65-0865-14) for 30 min. Cells were washed and stained with a combination of the following fluorophore-conjugated primary antibodies: rat anti-CD45-BV510 (clone 30-F11, BioLegend, #103137), rat anti-CD11b-PerCP/Cyanine5.5 (clone M1/70, BioLegend, #101227), rat anti-Ly6C-APC/Cyanine7 (clone HK1.4, BioLegend, #128025), and rat anti-Ly6G-eFluor^™^450 (clone 1A8-Ly6g, Thermo Fisher Scientific, #48-9668-82) at a 1:400 dilution for 30 min on ice. After an additional wash, cells were sorted by a BD FACSAria II using the 70 micron nozzle to sort single cells into 96-well plates containing mastermix (see below) with a sort speed of approximately 10,000/s. Raw flow cytometry data were analysed using FlowJo software (FlowJo LLC, Version 10.6.2). All scRNA-seq experiments were performed at the Single Cell Core Facility of the Sulzberger Columbia Genome Center, New York. Library preparation and RNA-sequencing was performed as described previously^77^. Briefly, cells were directly sorted into mastermix, containing 1x Maxima Reverse Transcriptase Buffer (Thermo Fisher Scientific, #EP0742), 40 U Maxima H Minus Reverse Transcriptase (Thermo Fisher Scientific, #EP0751), 4 U SuperaseIN (Thermo Fisher Scientific, #AM2694), 15% PEG (VWR, 97061-102), 1 μM TSO (Integrated DNA Technologies), and nuclease-free water. Template-switching reverse transcription was performed with adapter-linked oligo primers containing both cell- and molecule-specific barcodes (**Supplementary Table 5**). Excess primers were removed by adding 2 μL of Exonuclease I (Thermo Fisher Scientific, #EN0581) mix to each well and incubated at 37 °C for 30 min, 85 °C for 15 min, 75 °C for 30 s. All wells were then pooled into a single 15 mL conical tube and cDNA was purified and concentrated with Dynabeads MyOne Silane beads (Thermo Fisher Scientific, #37002D). The cDNA was split into duplicate reactions of 25 μL cDNA, 25 μL of 2× HIFI HotStart Ready Mix (Kapa Biosystems, #07958927001), and 0.2 M SMART PCR Primer (**Supplementary Table 5**). PCR was performed as described above. cDNA was purified with AMPure XP beads (Beckman Coulter, #A63880), visualized on an Agilent TapeStation and quantified with a Qubit II fluorometer (Thermo Fisher Scientific). Library preparation was performed using a modified protocol of the Nextera XT kit (Illumina, #FC-131-1024), purified twice with AMPure XP beads (Beckman Coulter, #A63880), and visualized and quantified as described above. Pooled, 3'-end libraries were sequenced on an Illumina NextSeq 500/550 apparatus. Reads were aligned to the mouse genome reference GRCm38 using STAR (version 2.5)^78^. Reads were assigned to cells and unique molecular identifiers (UMIs)^79^. The expression matrix for single cell data was processed using the package Seurat v3.1.5 in R^80^. Features for which fewer than 3 cells were detected were removed, effectively excluding unexpressed features. Cells having at least 1,000 and at most 4,000 features were retained. Cells with more than 5% of reads mapping to mitochondrial genes were discarded. The NormalizeData function was used to log-normalize the dataset with a scale factor of 10,000. The top 2,000 most variable features across cells were found using the function FindVariableFeatures. The ScaleData function was applied to scale the dataset. The variable features were used to carry out dimensional reduction using PCA. The optimal number of principal components to be used for dimensional reduction using Uniform Manifold Approximation and Projection (UMAP) was determined using ElbowPlot. FindNeighbors and FindClusters functions were utilized to construct a nearest neighbour graph and cluster cells in the dataset. UMAP was generated using the function DimPlot. The FindAllMarkers function was applied to determine markers for clusters in the UMAP plot. The FindMarkers function was used to carry out differential expression analysis for the three experimental groups.

### iDISCO+ staining, imaging and ClearMap analysis.

24 h after the SI test, *Ccr2*^rfp+/−^ mice were injected with 10% chloral hydrate and transcardially perfused with ice-cold 0.1 M PBS followed by 4% paraformaldehyde (PFA) (Electron Microscopy Sciences, #15713S). Intact brains were dissected out of the skull and postfixed in 4% PFA in PBS at 4 °C for 18 h. Brains were then cleared and stained according to the iDISCO+ staining protocol from http://www.idisco.info. The primary rabbit anti-RFP antibody (Rockland, #600-401-379, 1:1,000) and the corresponding secondary antibody (donkey anti-rabbit IgG, Alexa Fluor^™^ 647, Thermo Fisher Scientific, #A-31573, 1:1,000) were incubated with the brains for 7 days each at 37 °C. A LaVision lightsheet microscope with zoom body was used for sagittal half brain scanning with dynamic focus and a step size of 4 μm. Brain images were processed as previously described using ClearMap^39^. RFP^+^ cells were quantified using the cell detection module, with cell detection parameters optimized and validated based on the intensity and shape parameters of the signal. The autofluorescence channel was aligned to the Allen Institute’s Common Coordinate Framework using the Elastix toolbox. Brain areas were collapsed into their parent regions prior to analyses. RFP cell counts from each region were then correlated (Pearson correlation) with the SI score of defeated mice.

### *Ex vivo* electrophysiology.

Brains were rapidly extracted from isoflurane anaesthetized-mice, and coronal sections (250 μm) were sliced using a Compresstome (VF-210-0Z, Precisionary Instruments) in cold (0-4 °C) sucrose-based artificial cerebrospinal fluid (aCSF) containing: 87 mM NaCl (Sigma-Aldrich, #S7653), 2.5 mM KCl (Sigma-Aldrich, #P9333), 1.25 mM NaH_2_PO4 (Sigma-Aldrich, #71507), 4 mM MgCl_2_ (Sigma-Aldrich, #M2670), 0.5 mM CaCl_2_ (Sigma-Aldrich, #C8106), 23 mM NaHCO_3_ (Sigma-Aldrich, #S6297), 75 mM sucrose (Sigma-Aldrich, #S7903), 25 mM glucose (Sigma-Aldrich, #G7021). After 60 min in aCSF at 32 °C for recovery, slices were kept in oxygenated (95% O_2_ & 5% CO_2_) aCSF containing: 130 mM NaCl (Sigma-Aldrich, #S7653), 2.5 mM KCl (Sigma-Aldrich, #P9333), 1.2 mM NaH_2_PO_4_ (Sigma-Aldrich, #71507), 2.4 mM CaCl_2_ (Sigma-Aldrich, #C8106), 1.2 mM MgCl_2_ (Sigma-Aldrich, #M2670), 23 mM NaHCO_3_ (Sigma-Aldrich, #S6297), 11 mM Glucose (Sigma-Aldrich, #G7021) at room temperature for the rest of the day and individually transferred to a recording chamber continuously perfused at 2-3 mL/min with oxygenated aCSF. Patch pipettes (4-6 MΩ) were pulled from thin wall borosilicate glass using a micropipette puller (P-97, Sutter Instruments) and filled with a K-gluconate-based intra-pipette solution containing: 116 mM KGlu (Sigma-Aldrich, #P1847), 20 mM HEPES (Sigma-Aldrich, #H3375), 0.5 mM EGTA (Sigma-Aldrich, #E0396), 6 mM KCl (Sigma-Aldrich, #P9333), 2 mM NaCl (Sigma-Aldrich, #S7653), 4 mM ATP (Sigma-Aldrich, #A9187), 0.3 mM GTP (Sigma-Aldrich, #51120) (pH adjusted to 7.2 and osmolarity to 290 mOsm). Cells were visualized using an upright microscope with an IR-DIC lens and illuminated with a white light source (Scientifica). Excitability was measured in current-clamp mode by injecting incremental steps of current (0-300 pA, +20 pA at each step). For recording of spontaneous excitatory post-synaptic currents (sEPSCs), NAc medium spiny neurons (MSNs) were recorded from in voltage-clamp mode at −70 mV. Whole-cell recordings were performed using a patch-clamp amplifier (Axoclamp 200B, Molecular Devices) connected to a Digidata 1550 LowNoise acquisition system (Molecular Devices). Signals were low pass filtered (Bessel, 2 kHz) and collected at 10 kHz using the data acquisition software pClamp 11 (Molecular Devices). Electrophysiological recordings were extracted using Clampfit 11 (Molecular Devices) and analysed with R (version: 3.6.1, http://www.R-project.org). All groups were counterbalanced by days after CSDS. All recordings were performed while blinded to the experimental conditions.

### Human subjects.

Study participants with major depressive disorder (MDD) and healthy controls (HC), as assessed by the Structured Clinical Interview for the Diagnostic and Statistical Manual of Mental Disorders–Fifth Edition (SCID-5)^81^, were recruited through the Depression and Anxiety Center for Discovery and Treatment at the Icahn School of Medicine at Mount. The ISMMS review board approved the study, and written informed consent was obtained from all participants prior to any study procedure. Participants were compensated for their time and effort. Subjects provided demographic information and underwent a psychiatric evaluation using the SCID-5 conducted by trained study staff. Participants completed the Quick Inventory of Depressive Symptomatology-SR (QIDS-SR) to measure depressive symptom severity^82^. The Perceived Stress Scale^33^, a 10-item self-rating scale, was used to determine perceived stress levels. All participants underwent biochemistry and hematological laboratory testing, urine toxicology and pregnancy (if applicable) testing. At the time of enrollment, all participants were free of medications known to affect the immune system for at least two weeks. Participants were free of active infections or systemic illness. Subjects with concomitant unstable medical illnesses were excluded. Participants were free of current substances of abuse. On the day of blood draw, patients were fasted for at least 6 h. Blood was drawn into Vacutainer Gold Top 5 mL Silica Gel tubes (BD, #365968) for serum isolation and EDTA tubes (BD, #365975) to assess complete blood count and differential count (Sysmex XN-9100^™^ Automated Hematology System). For serum, blood was allowed to clot for > 30 min, then centrifuged at 1300 g for 15 min at 4 °C, then aliquoted and stored at −80 °C.

### Enzyme-linked immunosorbent assay (ELISA).

ELISAs were performed according to the manufacturer’s instructions (mouse MMP8: Abcam, #ab206982; human MMP8: R&D Systems, #DMP800B). For brain lysates, total protein was measured with the Pierce^™^ BCA Protein Assay Kit (Thermo Fisher Scientific, #23225). Plates were read on a SpectraMax 340PC384 microplate reader (Molecular Devices) and MMP8 or total protein levels were calculated from a serial dilution curve using SoftMax Pro 5 software (Molecular Devices).

### Multiplex assays.

Mouse plasma cytokines and chemokines were determined with the Milliplex MAP mouse cytokine/chemokine magnetic bead panel multiplex assay according to the manufacturer’s instructions (Millipore Sigma, #MCYTOMAG-70K), and mouse MMPs 2, 3, 9 and 12 were measured with Milliplex MAP Mouse MMP Magnetic Bead Panels 1 and 2 (Millipore Sigma, #MMMP1MAG-79K, #MMMP2MAG-79K).

### Transmission electron microscopy and image analysis.

Mice were injected with 10% chloral hydrate and transcardially perfused with 0.1 M sodium cacodylate buffer followed by ice-cold 2% PFA and post-fixed with 0.5% PFA at 4 °C. Tissue was sectioned on a vibratome, and freeze substitution and low temperature embedding of the specimens was performed as described previously^83,84,85^. Slices were cryoprotected by immersion in increasing concentrations of glycerol (from 10% to 30% in PBS) (v/v). Sections were plunged rapidly into liquid propane cooled by liquid nitrogen (−190 °C) in a Universal Cryofixation System KF80 (Reichert-Jung, Vienna, Austria). The samples were immersed in 1.5% uranyl acetate dissolved in anhydrous methanol (−90 °C, 24 h) in a cryosubstitution AFS unit (Leica, Vienna, Austria). The temperature was raised from −90 °C to −45 °C in steps of 4 °C/h. After washing with anhydrous methanol, the samples were infiltrated with Lowicryl HM20 resin (Electron Microscopy Sciences, Fort Washington, PA) at −45 °C. Polymerization with ultraviolet light (360 nm) was performed for 48 h at −45 °C, followed by 24 h at 0 °C. Ultrathin sections (80 nm) were cut with a diamond knife on a Leica UC7 ultramicrotome and mounted on 300 mesh copper grids using a Coat-Quick adhesive pen (Electron Microscopy Sciences). Images (n=10/animal) were taken using a Hitachi 7700 electron microscope (Hitachi High-Technologies Corporation America, Inc.) equipped with a XR81-B-M1-BT-FX, 8 Megapixel digital camera (Advanced Microscopy Techniques, Woburn, MA). Images were then imported into Adobe Photoshop (Adobe, 2022) and the extracellular space was manually scored using a computer tablet. Scoring was done by two independent investigators blinded to experimental conditions. Images were then imported into ImageJ (v1.53f51)^86^ and the percentage of marked area/total area was calculated.

### Immunohistochemistry and confocal microscopy.

Mice were injected with 10% chloral hydrate and transcardially perfused with ice-cold 0.1 M PBS (pH 7.4) followed by ice-cold 4% PFA (Electron Microscopy Sciences, #15713S). Intact brains were dissected out of the skull and postfixed in 4 % PFA at 4 °C for 18 h. Brains were then cryoprotected in 30% sucrose (Sigma, #S0389), frozen and sliced on a cryostat at 35 μm thickness. Sections were washed with PBS three times and incubated in blocking solution (3% normal donkey serum (Jackson Immuno Research, #017-000-121), 0.3% Triton X-100 (Sigma, T9284) in PBS) for 2 h. Sections were then incubated in primary antibodies (rat anti-CD31, 1:300, Biolegend, #102501; rabbit anti-RFP, 1:300, Rockland, #600-401-379) overnight at 4 °C. The next day, sections were washed in PBS with 0.3% Tween-20 (PBST, [Sigma, P7949]) three times for 15 min each, then incubated with anti-rabbit-Cy2 and anti-rat-Cy5 secondary antibodies for 2 h (1:400, Jackson Immunoresearch, #711-225-152, #712-175-153, respectively). Sections were washed again three times with PBST. Slices were then mounted on slides, air-dried overnight, dehydrated, and coverslipped with DPX (Electron Microscopy Sciences, #13510). All slices were imaged using a Zeiss LSM 780 confocal microscope. 3D reconstruction was performed with the IMARIS software (v9.9) (Oxford Instruments Group).

### Biotinylation.

Biotinylation of mouse recombinant MMP8 (rMMP8) (Bio-techne, #2904-MP-010) was performed using the EZ-Link^™^ Sulfo-NHS-Biotin kit according to the manufacturer’s instructions (Thermo Fisher Scientific, #A39256). Biotinylated rMMP8 was separated from unbound biotin using Pierce^™^ C18 Spin Columns, 7K MWCO, (Thermo Fisher Scientific, #89870), which recovers proteins and macromolecules larger than 7 kDa. Biotinylated rMMP8 was injected retro-orbitally into anaesthetized mice. After 2 h of circulation, mice were euthanized and perfused with ice-cold PBS followed by 4% PFA. Brain tissue processing and imaging was performed as described in the Immunohistochemistry and confocal microscopy section, with the following antibodies: Biotin was visualized using the Oregon Green^®^ 488 conjugate of NeutrAvidin^®^ biotin-binding protein (Thermo Fisher Scientific, #A6374). Counterstaining was performed using rabbit anti-NeuN (1:500, Abcam, #ab177487) and rat anti-CD31 (1:300, Biolegend, #102501).

### Statistical analysis.

Detailed statistical information for each experiment can be found in **Supplementary Table 2**. Unless described otherwise, statistical analyses were performed with GraphPad Prism software (Version 9, GraphPad Software Inc.) or SPSS version 24 (IBM Corp., SPSS Inc., Chicago IL, USA). Outliers were identified using the Grubbs's test and excluded from statistical analyses. Level of statistical significance was set at p < 0.05.

## Extended Data

## Data Availability

RNA-seq data have been deposited in the Gene Expression Omnibus under accession number (GSE ID: GSE202662, reviewer token: wvwdoiicjjcjbyt). All the other data are available from the corresponding author upon reasonable request.
